# Intranasal administration of allergen increases specific IgE whereas intranasal omalizumab does not increase serum IgE levels—A pilot study

**DOI:** 10.1111/all.13343

**Published:** 2017-12-12

**Authors:** J. Eckl‐Dorna, R. Fröschl, C. Lupinek, R. Kiss, P. Gattinger, K. Marth, R. Campana, I. Mittermann, K. Blatt, P. Valent, R. Selb, A. Mayer, K. Gangl, I. Steiner, J. Gamper, T. Perkmann, P. Zieglmayer, P. Gevaert, R. Valenta, V. Niederberger

**Affiliations:** ^1^ Department of Otorhinolaryngology Medical University of Vienna Vienna Austria; ^2^ Clinical Institute for Laboratory Medicine Medical University of Vienna Vienna Austria; ^3^ Division of Immunopathology Department of Pathophysiology and Allergy Research Center for Pathophysiology, Infectiology and Immunology Medical University of Vienna Vienna Austria; ^4^ Division of Hematology and Hemostaseology Department of Internal Medicine I Medical University of Vienna Vienna Austria; ^5^ Center for Medical Statistics, Informatics, and Intelligent Systems Section for Medical Statistics Medical University of Vienna Vienna Austria; ^6^ Vienna Challenge Chamber Vienna Austria; ^7^ Upper Airway Research Laboratory (URL) Ghent University Hospital Ghent Belgium

**Keywords:** allergen, half‐life, IgE, intranasal challenge, omalizumab

## Abstract

**Background:**

Administration of the therapeutic anti‐IgE antibody omalizumab to patients induces strong increases in IgE antibody levels.

**Objective:**

To investigate the effect of intranasal administration of major birch pollen allergen Bet v 1, omalizumab or placebo on the levels of total and allergen‐specific IgE in patients with birch pollen allergy.

**Methods:**

Based on the fact that intranasal allergen application induces rises of systemic allergen‐specific IgE, we performed a double‐blind placebo‐controlled pilot trial in which birch pollen allergic subjects were challenged intranasally with omalizumab, placebo or birch pollen allergen Bet v 1. Total and allergen‐specific IgE, IgG and basophil sensitivity were measured before and 8 weeks after challenge. For control purposes, total, allergen‐specific IgE levels and omalizumab‐IgE complexes as well as specific IgG levels were studied in subjects treated subcutaneously with either omalizumab or placebo. Effects of omalizumab on IgE production by IL‐4/anti‐CD40‐treated PBMCs from allergic patients were studied in vitro.

**Results:**

Intranasal challenge with Bet v 1 induced increases in Bet v 1‐specific IgE levels by a median of 59.2%, and this change differed significantly from the other treatment groups (*P* = .016). No relevant change in allergen‐specific and total IgE levels was observed in subjects challenged with omalizumab. Addition of omalizumab did not enhance IL‐4/anti‐CD40‐induced IgE production in vitro. Significant rises in total IgE (mean IgE before: 131.83 kU/L to mean IgE after: 505.23 kU/L) and the presence of IgE‐omalizumab complexes were observed after subcutaneous administration of omalizumab.

**Conclusion:**

Intranasal administration of allergen induced rises of allergen‐specific IgE levels, whereas intranasal administration of omalizumab did not enhance systemic total or allergen‐specific IgE levels.

**Abbreviations**
BCR: B‐cell receptor
BSA: Bovine serum albumin
Cε3: Constant region 3
CI: Confidence interval
ELISA: Enzyme‐linked immunosorbent assay
FCS: Foetal calf serum
FcεRI: High‐affinity receptor for IgE
FcRn: Neonatal Fc receptor
GMP: Good manufacturing practice
IgE: Immunoglobulin E
IL: Interleukin
ISU: International standard units
kU/L: Kilo units per litre
kUA/L: Kilo units antigen per litre
mAb: Monoclonal antibody
MFI: Mean fluorescence intensity
PBMC: Peripheral blood mononuclear cell
PBS: Phosphate‐buffered saline
RBL: Rat basophilic leukaemia
s.c.: Subcutaneous

## 1 INTRODUCTION

Immunoglobulin E (IgE) is the key antibody class responsible for allergic symptoms in sensitized patients.^1^ Cross‐linking of IgE bound to the surface of mast cells and basophils via its high‐affinity receptor (FcεRI) results in degranulation of the cells and the release of inflammatory mediators, cytokines and proteases.^2^, ^3^, ^4^

In order to prevent IgE binding to its receptors, omalizumab—a monoclonal humanized anti‐IgE antibody that specifically binds to the constant region 3 (Cε3) of IgE—has been developed and has been shown to be effective for the treatment of a variety of allergic manifestations and in particular for severe IgE‐mediated asthma^.^ 5, 6, 7 As the binding site of omalizumab overlaps with the binding site of IgE to FcεRI, omalizumab does not induce mast cell and basophil activation or anaphylactic reactions and inhibits binding of circulating IgE to its receptor on effector cells. Administration of omalizumab therefore reduces mediator release after approximately 16 weeks of therapy, which is associated with a downregulation of FcεRI expression on basophils, mast cells and antigen‐presenting cells.^8^, ^9^, ^10^, ^11^ More recently, it has been shown that omalizumab is also effective for the treatment of chronic spontaneous urticaria.^12^

Omalizumab targets the Cε3 region of IgE, which is considered to be accessible also on membrane‐bound IgE in the form of B‐cell receptors (BCRs) on IgE^+^ memory B cells. It is therefore possible that cross‐linking of the BCR on IgE^+^ memory cells may contribute to the strong increases in IgE levels which are observed in patients treated with omalizumab in addition to a prolongation of the half‐life of IgE within the omalizumab‐IgE immune complexes.^5^, ^13^, ^14^ In this context, it has recently been hypothesized that the binding of omalizumab to IgE may prevent degradation of IgE via binding of the resulting IgE‐IgG complex to the neonatal Fc receptor (FcRn) and thus protecting it from endolysosomal degradation or that the IgE‐omalizumab complexes do not undergo pinocytosis.^15^ The other hypothesis that cross‐linking of the BCR by omalizumab may result in increased IgE production is supported by the finding that injection of anti‐IgE in previously primed mice was driving a secondary IgE response^16^ and that secondary IgE responses in mice could also be boosted by repetitive B‐cell epitopes which also may have cross‐linked the BCR.^17^ In the nasal mucosa of allergic patients, the presence of IgE^+^ B cells as well as plasma cells^18^ and local IgE production^19^ have been demonstrated. In addition, a strong rise of systemic allergen‐specific IgE has been observed in subjects after nasal administration of allergens^20^, ^21^, ^22^, ^23^ indicating the potential presence of memory IgE^+^B cells in the nasal mucosa. We therefore investigated the effect of intranasal administration of omalizumab, allergen or placebo on allergen‐specific and total systemic IgE. We applied in a double‐blind, placebo‐controlled clinical study omalizumab to birch pollen allergic subjects intranasally and, as positive and negative control, included allergic subjects who received intranasal allergen challenge and buffer, respectively. The effects of intranasal omalizumab and allergen exposure on total, allergen‐specific IgE and IgG levels as well as on basophil sensitivity were then analysed for a period of 8 weeks. In addition, we studied the effects of subcutaneous (s.c.) omalizumab administration in allergic subjects regarding changes in total, allergen‐specific IgE and allergen‐specific IgG levels.

## 2 MATERIAL AND METHODS

### 2.1 Study design for intranasal challenge with omalizumab, Bet v 1 or placebo

**Figure 1:**
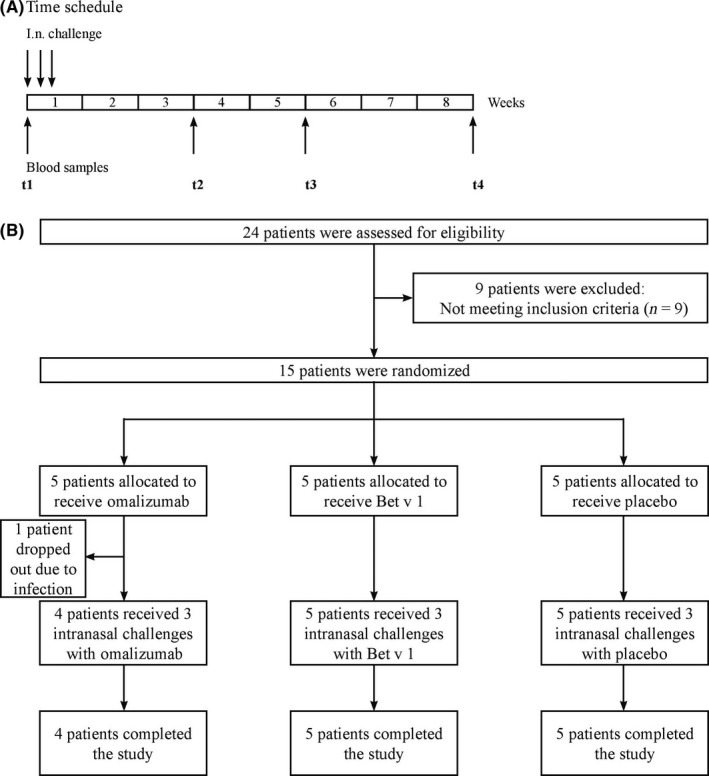
Design of the intranasal challenge study. A, Subjects were challenged intranasally with either omalizumab, Bet v 1 or placebo on 3 consecutive days (d1‐d3, arrows, top). Blood samples (arrows, bottom) were taken before the first (t1) challenge and on days 21 (t2), 35 (t3) and 56 (t4) (±4 days) after the first challenge. B, Flow chart depicting number of subjects who were screened (n = 24), randomized (n = 15), and who completed (n = 14) the study

The study was performed over 8 weeks outside of the birch pollen season (November to January) as an investigator‐initiated, randomized, double‐blind and placebo‐controlled pilot study. The study was approved by the Ethical committee of the University of Vienna and Austrian authorities and registered in the European Clinical Trials Database (EudraCT: 2012‐004193‐25) and in the ClinicalTrial.gov data base (NCT03019237). During the screening visit, 24 subjects between 18 and 60 years of age were assessed (Figure 1). Fifteen subjects fulfilled all inclusion criteria and were thus selected for the study. Those subjects were suffering from moderate‐to‐severe allergic rhinitis to birch pollen for at least 2 seasons according to medical history. Birch pollen allergy was confirmed by skin prick test (ALK‐Abello, Wedel, Germany) and measurement of Bet v 1‐specific IgE antibodies in serum (ImmunoCAP, Phadia, Uppsala, Sweden). Only patients with Bet v 1‐specific IgE levels higher than 3.51 kUA/l who were not previously exposed to omalizumab were included in the study. The absence of nasal polyps or substantial deviation of the nasal septum was confirmed by anterior rhinoscopy prior to nasal challenge. After the screening visit, patients were randomized into 3 groups at a ratio of 1:1:1 (5 patients per group, using an online randomization programme provided by the Centre of Medical Statistics, Informatics, and Intelligent Systems of the Medical University of Vienna) for intranasal challenge with either omalizumab (Xolair^®^, Novartis Pharmaceuticals Corporation, New York, NY), Bet v 1 (Biomay, Vienna, Austria) or placebo (0.9% sodium chloride solution). One patient in the omalizumab challenge group withdrew from the study due to illness (upper respiratory tract infection) just before the first nasal challenge. At visit 1 (t1), blood samples were taken and the first nasal challenge was performed. Two more nasal challenges were performed on the consecutive 2 days. Further blood samples were taken 3 (t2), 5 (t3) and 8 (t4) weeks (±4 days) after the first challenge. The clinical investigators and study subjects were blinded regarding the administered substances. Randomization and preparation of the study drugs (omalizumab, Bet v 1 or placebo) were carried out by a person who was not involved in the study and had no contact with the subjects to ensure the double‐blinded nature of the study.

### 2.2 Nasal allergen challenge

Purified recombinant Bet v 1 produced according to good manufacturing practice (GMP) (Biomay AG) or Xolair (Novartis) was diluted in sterile 0.9% sodium chloride freshly before use. Bet v 1 was applied at a concentration of 50 μg/mL as this concentration has previously been shown to lead to increase in systemic serum IgE levels after intranasal administration.^21^ Omalizumab was applied at an equimolar concentration (438 μg/mL), and 0.9% sodium chloride was applied as placebo. Patients were administered 1 puff per nostril using a metered pump delivering 15 μL per puff.

### 2.3 Sera from subjects who had received s.c. administration of omalizumab or placebo

Recruitment, study population, administration of the drug as well as dosage and blood draw schedule are described in detail in Gevaert et al.^24^ Sera from this study were obtained and tested in our study as follows: sera “Before s.c. administration” were tested at visit V1 (V1: screening visit, at least 2 weeks before visit V2 where first dose of omalizumab was administered) except for subject S9 where serum was from V2. Sera “After s.c. administration” were tested at visit V8 (12 weeks after first subcutaneous administration) except for subject S18 where serum from visit V6 (8 weeks after first subcutaneous administration) and for subject S16 where serum from visit V10 (16 weeks after first subcutaneous administration) was tested. Skin prick tests were performed using a grass mix, 2 different tree mixes (*Betulaceae* and *Fagaceae*), as well as mugwort, mould, cat, dog, D. pter., D. far. extract all purchased from Stallergenes (Antony, France).

### 2.4 Measurements of total IgE, allergen‐specific IgE and allergen‐specific IgG levels in sera

Total IgE and IgE specific to birch extract (t3), Bet v 1 (t215), grass (g6), olive (t9), cat (e1) and house dust mite (d1) were measured by ImmunoCAP (Phadia, Uppsala, Sweden). Allergen‐specific IgG levels were analysed using a microarray based on ImmunoCAP ISAC technology (Phadia‐Thermofisher, Uppsala, Sweden) containing more than 170 different allergen molecules^25^ according to the manufacturer's instruction.

### 2.5 Basophil activation

Allergen‐induced basophil activation was determined in heparinized blood samples obtained before and after intranasal challenge by measuring the upregulation of CD203c expression on basophils upon exposure to rBet v 1 (0.01 μg/mL), anti‐IgE (1 μg/mL) or buffer (phosphate‐buffered saline = PBS). Mean fluorescence intensities of stimulated (MFIstim) and unstimulated (MFIcontrol) cells were determined for triplicate cultures by flow cytometry, and the upregulation of CD203c expression was expressed as stimulation index (MFIstim: MFIcontrol) (SI).^26^

In addition, basophil activation tests were performed by passive loading of rat basophilic leukaemia (RBL) cells expressing the α/β/γ subunits of the human FcεRI^27^ with 1:20 diluted sera from subjects obtained at different time points (t1 and t3). Degranulation was induced by addition of rBet v 1 (0.01 μg/mL) or buffer as control. Released β‐hexosaminidase was measured, and percentage release was calculated as described.^28^ All measurements were performed in triplicates. Percentage changes in β‐hexosaminidase release between t1 and t3 were calculated.

### 2.6 ELISA measurement of omalizumab‐IgE complexes in serum

ELISA plates (ie, 384 well plates, Nunc Maxisorp, Roskilde, Denmark) were coated with 1 μg/mL human anti‐omalizumab (drug/target complex) HCA238 (Bio‐Rad, Hercules, CA) overnight at 4°C and blocked with PBS/5% v/v BSA/0.05% v/v Tween‐20(blocking buffer). As a standard, purified monoclonal chimeric Bip 1 IgE (5 μg/mL)^29^ was incubated with varying concentrations of omalizumab in PBS containing 10% v/v human serum (Lonza Group LTD, Basel, Switzerland) as well as 0.05% v/v Tween‐20. Chimeric Bip 1 IgE standard and omalizumab were complexed at room temperature for 1 hour before applying the standards to the plates. Samples were diluted 1:10 in PBS with 0.05% v/v Tween‐20. Standards and samples to be analysed were added to plates and incubated at 4°C overnight. After washing, the HRP‐conjugated human anti‐omalizumab antibody (drug/target complex) HCA237P (2 μg/mL, Bio‐Rad, Hercules, CA), diluted in HISPEC buffer (Bio‐Rad, Hercules, CA), was applied to the plates for 1 hour at RT. After thorough washing, tetramethylbenzidine substrate (Ebioscience, San Diego, CA, USA) was applied to the plates for the enzymatic colour reaction and stopped by addition of 1m phosphoric acid_._ OD levels were read at 450 nm. Measurements were performed in triplicates, and background was subtracted.

### 2.7 Isolation and cultivation of peripheral blood mononuclear cells (PBMCs)

Heparinized blood samples were obtained from 2 birch and grass pollen allergic subjects after written informed consent was given (approved by the Ethical committee of the Medical University of Vienna, EK1641/2014). PBMCs were isolated from heparinized blood samples of allergic patients using Ficoll density gradient separation (Amersham Biosciences AB, Uppsala, Sweden). Cells were washed 3 times in phosphate‐buffered saline (PBS) (PAA Laboratories, Pasching, Austria) and then cultured in RPMI 1640 containing L‐glutamine (BioWhittaker, Verviers, Belgium), supplemented with 10% foetal calf serum (FCS) (PAA Laboratories, Pasching, Austria) and 50 μg/mL gentamicin (Sigma, St. Louis, MO, USA). Cells were either left unstimulated or stimulated with IL‐4 (400 IU/mL, R&D, Minneapolis, MI, USA) and anti‐CD40 (clone G28.5, 1 μg/mL Biolegend, San Diego, CA, USA) in the presence or absence of omalizumab (3 μg/mL) or isotype control (3 μg/mL, Southern Biotech, Alabama, AL). Supernatants were taken after 1 week of culture and frozen at −20°C until measurement of total IgE levels by ImmunoCAP (total IgE low range, Phadia‐Thermofisher, Uppsala, Sweden). Percentage change in total IgE levels compared to supernatants from unstimulated cells was calculated.

### 2.8 Statistics

Statistical analyses were carried out with SAS 9.4. (SAS Institute Inc., Cary, NC, USA) or R 3.3.1 (The R Foundation for Statistical Computing, Vienna, Austria). For all analyses, the significance level was set to 0.05. To analyse the percentage change among the intranasal treatment groups, the Kruskal‐Wallis test was conducted. Only if the *P*‐value of the Kruskal‐Wallis test was < .05, the two‐sided Wilcoxon rank‐sum test was calculated for pairwise group comparison. For both, the Kruskal‐Wallis test and the Wilcoxon rank‐sum test, exact *P*‐values were computed. To analyse the difference before and after subcutaneous administration of omalizumab, paired *t* tests and 95% confidence intervals (CI) for the mean difference of visits were calculated for each treatment group separately, assuming a normal distribution of the data. To assess correlation between total IgE levels and omalizumab‐IgE complexes, Pearson correlation coefficients, 95% CIs as well as exact *P*‐values were calculated. Due to the exploratory nature of this pilot study, no correction for multiple testing was applied.

## 3 RESULTS

### 3.1 Design of a study to investigate the effects of intranasal application of omalizumab on systemic IgE levels

Allergic patients suffering from birch and grass pollen allergy (n = 15) (Table 1, Table S1) included in the intranasal challenge study were randomized into 3 groups (5 patients each) for intranasal challenge with omalizumab, Bet v 1 or placebo (Table 1, Figure 1). A group challenged intranasally with Bet v 1 was included as a positive control because it has been shown that intranasal challenge with Bet v 1 induces systemic rises of Bet v 1‐specific IgE levels.^20^, ^21^, ^22^, ^23^ One subject of the omalizumab group dropped out 1 day before intranasal challenge due to a viral upper respiratory tract infection. All other participants completed the study. Demographic, clinical and serological data of the subjects are displayed in Tables 1 and Table S1 indicating a balanced distribution of mean Bet v 1‐specific and mean total IgE levels in the 3 groups.

**Table 1:** Summary of the demographic and clinical characterization of study subjects

Characteristic	i.n. challenge groups		
	Omalizumab (n = 4)	Bet v 1 (n = 5)	Placebo (n = 5)
Male/Female	0/4	2/3	1/4
Age (y)			
Mean ± SD	23.5 ± 2.7	28.4 ± 6.2	28.4 ± 9.9
Range	21‐27	22‐35	22‐46
Allergic symptoms ‐no (%)			
Rhinitis	4 (100)	5 (100)	5 (100)
Conjunctivitis	3 (75)	5 (100)	5 (100)
Atopic dermatitis	0 (0)	2 (40)	5 (100)
Total IgE (kU/L)			
Mean ± SD	449 ± 440	285 ± 193	429 ± 262
Range	61.4‐954	101‐549	129‐699
Bet v 1‐spec. IgE (kUA/L)			
Mean ± SD	24.7 ± 21.7	22.9 ± 15.7	29.8 ± 10.6
Range	9.4‐55.5	5.5‐43.9	12.2‐39.6

In addition, we investigated sera obtained from subjects before and after receiving omalizumab (n = 15) or placebo (n = 6) subcutaneously^24^ because it has been shown that subcutaneous injection of omalizumab induces rises of total serum IgE. These patients have been previously described by Gevaert et al,^24^ and their demographic and clinical characteristics are listed in Table S2.

### 3.2 Intranasal challenge with Bet v 1 increases allergen‐specific IgE levels while intranasal omalizumab challenge has no effect on allergen‐specific or total IgE levels

**Figure 2:**
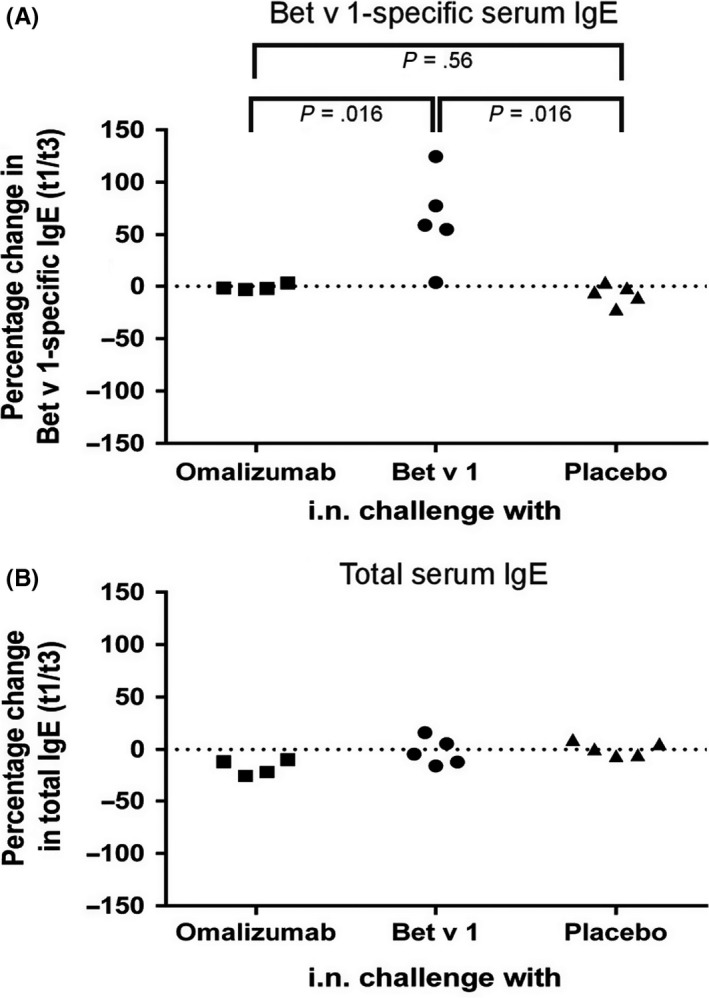
Percentage change in total and Bet v 1‐specific serum IgE levels following intranasal challenge with omalizumab, Bet v 1 or placebo. Percentage changes (y‐axes) of (A) Bet v 1‐specific and (B) total IgE levels between t1 (before challenge) and t3 (day 35 ± 4 days after challenge) are shown for each subject. Significant differences between the treatment groups were observed only for Bet v 1‐specific IgE (Kruskal‐Wallis test: *P* = .0053) levels but not for total IgE levels (Kruskal‐Wallis test: *P* = .0521). Pairwise comparisons using the two‐sided Wilcoxon rank‐sum test were therefore only performed for Bet v 1‐specific IgE levels and *P* values are indicated

Subjects who were challenged intranasally with Bet v 1 on 3 consecutive days outside of the birch pollen season showed significant increases in Bet v 1‐specific serum IgE levels as compared to subjects who were challenged with placebo or omalizumab (Kruskal‐Wallis test: *P* = .0053, two‐sided Wilcoxon rank‐sum test Bet v 1 vs omalizumab and Bet v 1 vs placebo: *P* = .016) (Figure 2A, Table S3). The median increase in Bet v 1‐specific serum IgE observed at t3 in the Bet v 1‐challenged group was 59.23% (range: 3.55%‐124.60%). No relevant changes in total or Bet v 1‐specific IgE levels were observed after nasal challenge with omalizumab, and there were no statistically significant differences in changes of total IgE levels between t1 and t3 between the 3 treatment groups (Kruskal‐Wallis Test: *P* = .0521) (Figure 2B). There were also no relevant changes observed for grass, olive, cat or house dust mite‐specific IgE levels between t1 and t3 between the 3 groups (Table S3).

### 3.3 Loading of basophils with sera after Bet v 1 challenge increases sensitivity but patient's basophil sensitivity is not increased

**Figure 3:**
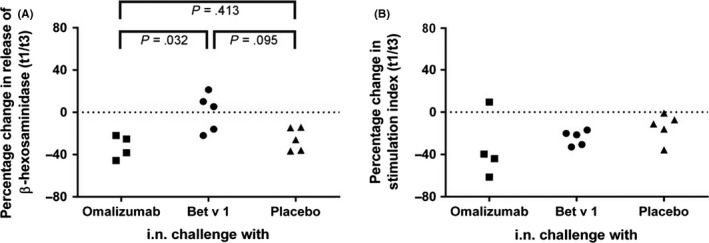
Effects of changes of allergen‐specific IgE levels on basophil activation. (A) Rat basophilic leukaemia (RBL) cells expressing human FcεRI were loaded with sera obtained at t1 and t3 and then stimulated with Bet v 1. Percentage changes (y‐axes) of β‐hexosaminidase release between t3 and t1 are shown. (B) Peripheral mononuclear cells (PBMCs) from subjects challenged intranasally (*x*‐axis: omalizumab, Bet v 1 or placebo) were stimulated with Bet v 1. Differences in the stimulation indices for the upregulation of CD203c between t3 and t1 are shown as percentage changes (y‐axes). Significant differences between the 3 treatment groups were observed for the RBL assay (Kruskal‐Wallis test: *P* = .0463) shown in (A) but not for the assays using basophils directly collected from subjects (Kruskal‐Wallis test: *P* = .1934) shown in (B). Pairwise comparisons using the two‐sided Wilcoxon rank‐sum test were therefore only performed for the RBL assay and *P*‐values are indicated

In a first set of experiments, we investigated whether loading of RBL cells expressing the human FcεRI with sera from subjects obtained 5 weeks after intranasal challenge with Bet v1 increases Bet v 1‐specific sensitivity. We found significant group differences between the intranasally challenged groups (Kruskal‐Wallis: *P* = .0463). The sensitivity of basophils loaded with sera from Bet v 1‐challenged subjects was significantly higher than that of basophils loaded with sera from omalizumab‐challenged subjects (pairwise Wilcoxon test: *P* = .032) (Figure 3A). The sensitivity of basophils loaded with sera from Bet v 1‐challenged subjects was also greater than that of basophils loaded with serum from placebo‐challenged subjects but did not reach significance (*P* = .095).

No differences regarding Bet v 1‐specific sensitivity was found when basophils from challenged subjects were obtained at t1 and t3 and compared in vitro for allergen‐specific sensitivity (Figure 3B). Basophil activation in PBMCs isolated from all subjects was measured by assessing CD203c upregulation by flow cytometry upon addition of Bet v 1 to cells. The change between t1 and t3 was calculated, but no relevant differences in basophil sensitivity were observed between the treatment groups (Kruskal‐Wallis test: *P* = .1934) (Figure 3B).

### 3.4 Subcutaneous application of omalizumab induces a rise in total IgE and the presence of IgE‐omalizumab complexes in the serum

**Figure 4:**
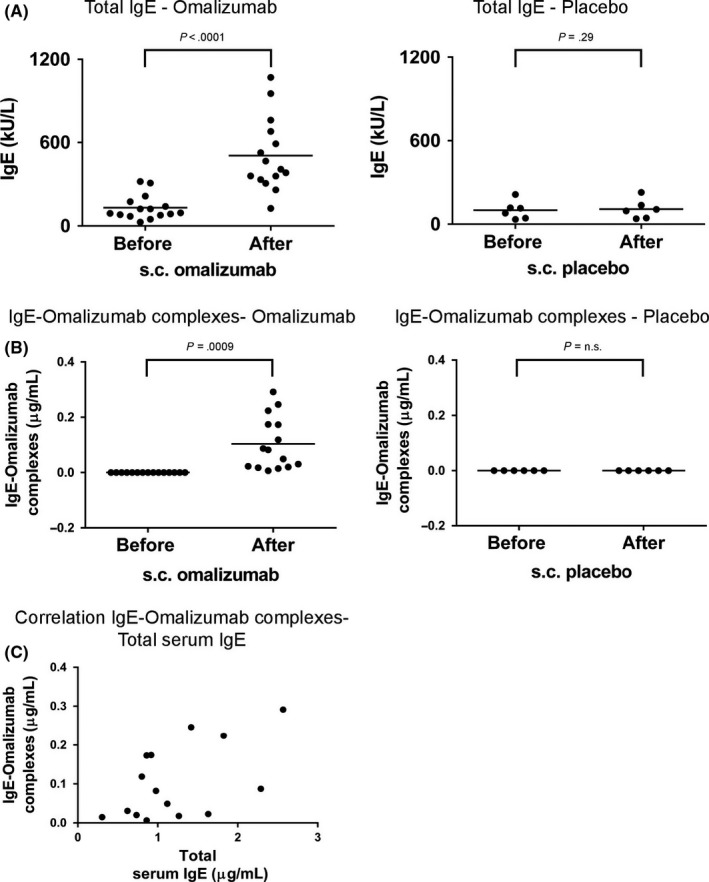
Total serum IgE and IgE‐omalizumab complex levels in subjects after subcutaneous omalizumab administration. (A) Total serum IgE levels (*y*‐axis: kU/L) and (B) levels of IgE‐omalizumab complexes (*y*‐axis: μg/mL) in subjects before and after subcutaneous administration of omalizumab (left, n = 16) or placebo (right, n = 6). Sera “Before s.c. administration” were obtained at visit V1 (V1: screening visit, at least 2 weeks before visit V2 where first dose of omalizumab was administered) except for subject S9 where serum was obtained at V2. Sera “After s.c. administration” were obtained at visit V8 (12 weeks after first subcutaneous administration) except for subject S18 where serum obtained at visit V6 (8 weeks after first subcutaneous administration) and for subject S16 where serum was obtained at visit V10 (16 weeks after first subcutaneous administration). (C) Correlation of total IgE levels (*x*‐axis) with IgE‐omalizumab complexes (*y*‐axis) is displayed for patients after subcutaneous omalizumab administration. Significant results (*P*‐values) are indicated

The analysis of sera from subjects who had received subcutaneous administration of omalizumab (n = 15) or placebo (n = 6)^24^ showed a significant rise in mean total IgE levels, that is mean 131.83 kU/L before to 505.23 kU/L, after administration of omalizumab (mean difference [95% CI]: 373.40 [264.23; 482.58], *P* < .0001, n = 15) (Figure 4A). In patients who had received placebo s.c., no significant differences regarding IgE levels before and after application were observed (mean difference [95% CI]: 7.86 [‐9.10; 24.81], *P* = .29, n = 6).

When we analysed allergen‐specific IgE levels, a fourfold to sevenfold rise in allergen‐specific IgE was observed after subcutaneous administration of omalizumab but not of placebo regardless of the nature of the tested allergen (Table S4). Interestingly, in 3 patients, allergen‐specific IgE rose over the cut‐off level of 0.35 kUA/L for IgE measurements after subcutaneous omalizumab administration (ie, cat‐specific IgE: subject S6: 0.28 (before treatment) → 2.07 (after treatment) kUA/L; house dust mite‐specific IgE: subject S7: 0.11 → 0.43 kUA/L and subject S9: 0.11 → 0.37 kUA/L). As already reported in another study, it seems that administration of omalizumab can reveal an already existing IgE sensitization below the cut‐off levels of IgE tests.^13^

Using an ELISA based on antibodies recognizing specifically IgE‐omalizumab complexes but neither noncomplexed IgE nor noncomplexed omalizumab alone, omalizumab‐IgE complexes were detected in the sera from all subjects after subcutaneous omalizumab administration. The levels of IgE‐omalizumab complexes rose significantly from 0 μg/mL before to an average of 0.104 μg/mL in subjects receiving omalizumab (Figure 4B: mean difference [95% CI]: 0.104 [0.051; 0.157], *P* = .0009, n = 15). A statistically significant correlation between serum IgE levels and the amount of IgE‐omalizumab complexes was noted; however, the corresponding confidence interval is wide due to the low sample size (Figure 4C: Pearson correlation coefficient [95% CI]: 0.54 [0.04; 0.83], *P* = .0359, n = 15).

### 3.5 Neither intranasal nor subcutaneous application of omalizumab affects allergen‐specific IgG levels

We also analysed allergen‐specific IgG levels in sera obtained from subjects before and after intranasal or subcutaneous application of omalizumab using a microarray containing more than 170 different allergens.^25^ There were no consistent and no significant changes in allergen‐specific IgG levels after intranasal or after subcutaneous application of omalizumab, Bet v 1 or placebo for Bet v 1, Phl p 1, Phl p 5, Ole e 1, Fel d 1 and Der p 1 (Table S5; Fig. S1).

### 3.6 Omalizumab does not increase IgE production induced in allergic patients' PBMCs stimulated with anti‐CD40/IL‐4 in vitro

To further investigate the potential effect of omalizumab on IgE production in vitro, we stimulated PBMCs derived from allergic subjects (Subject 1 and 2) with anti‐CD40 and IL‐4 in the presence or absence of omalizumab as well as an isotype control for omalizumab. Anti‐CD40 and IL‐4 stimulation induced an increase in total IgE levels in culture supernatants of both allergic subjects as previously described^30^, ^31^ (Fig. S2). Addition of omalizumab had no relevant effect on IgE production in the PBMC cultures.

## 4 DISCUSSION

In the present study, we investigated the effect of intranasal administration of Bet v 1, omalizumab and placebo on the levels of total and allergen‐specific IgE in patients with birch pollen allergy. The pilot clinical trial performed by intranasal administration of Bet v 1 and omalizumab was based on the observation that intranasal administration of allergen to allergic patients induced a robust increase in allergen‐specific IgE antibodies, eventually by targeting IgE‐producing cells.^20^, ^21^, ^22^, ^23^ In fact, histological analyses of nasal mucosal biopsies have revealed the presence of IgE^+^ B cells and plasma cells in the nasal mucosa.^18^ Furthermore, Sε switch circles have been found in nasal biopsies after ex vivo allergen challenge suggesting local synthesis of IgE in the nasal mucosa.^19^ We therefore hypothesized that IgE‐producing B cells in the nasal mucosa are the source of the allergen‐induced systemic IgE rises and can be reached and stimulated via the intranasal route. Indeed, we were able to demonstrate that nasal administration of the major birch pollen allergen Bet v 1 led to a significant increase in Bet v 1‐specific IgE levels 5 weeks after the challenge. Interestingly, these increases in Bet v 1‐specific IgE levels observed after 5 weeks did not lead to increased basophil sensitivity when basophils of allergic patients were exposed to the Bet v 1 allergen. In contrast, when we passively sensitized basophils with serum taken at the same time, an increased Bet v 1‐specific basophil sensitivity was observed.

There are several mutually not exclusive explanations why there are differences regarding allergen‐specific sensitivity when testing allergic patients basophils isolated at different time points from the patients vs testing cultured basophils. First, it is possible that the intrinsic sensitivity of the patient's basophils may vary at different time points. Second, testing of patients basophils at different time points may introduce variation. Third, experiments with patient's basophils are carried out in the presence of allergen‐specific IgG. In fact, allergen‐specific IgG increases were noted in several serum samples which may have affected allergen‐induced basophil activation (Table S5). Finally, it is possible that at the time points investigated (ie, t1 and t3) the newly produced IgE was not yet fully loaded onto the patients basophils which were preoccupied with the IgE which was there before.

The results can thus be summarized as follows: intranasal application of the major birch pollen allergen Bet v 1 led to an increase in allergen‐specific but not total systemic IgE levels, while intranasal omalizumab application had no effect on total or allergen‐specific IgE levels. Basophil sensitivity in patients did not change after challenge with the allergen despite a significant increase in Bet v 1‐specific IgE levels. In contrast, rat basophilic leukaemia cells expressing the human FcεRI which had been passively sensitized with the sera from allergen‐challenged patients showed increased allergen sensitivity after the challenge.

Furthermore, our in vitro experiments showed that omalizumab had no effect on the secretion of IgE ex vivo using PBMCs primed with IL‐4 and anti‐CD40. In patients having undergone subcutaneous omalizumab administration, a significant rise in systemic IgE levels and the presence of IgE‐omalizumab complexes were demonstrated. In summary, these data suggest that the rise of systemic IgE levels observed in omalizumab‐treated patients is most likely due to the formation of IgE‐omalizumab complexes increasing the half‐life of IgE and not due to an increase in IgE production by IgE^+^ B cells.

We have thus addressed two mutually nonexclusive possibilities to explain the rises of IgE levels in allergic patients undergoing therapy with the monoclonal anti‐IgE antibody omalizumab. The first possibility is based on findings that the administration of anti‐IgE antibodies into primed mice^16^ as well as injection of oligomeric allergen derivatives^17^ which are able to cross‐link the BCR on IgE‐producing memory cells into sensitized mice can boost secondary IgE production. This hypothesis has been controversially discussed with arguments in favour^14^, ^16^, ^32^, ^33^ and against^34^, ^35^ the potential of omalizumab to activate IgE^+^ memory B cells. In the present study, both in our in vivo intranasal challenge model and in our in vitro approach using a well‐established system to induce IgE production,^30^, ^31^ we did not observe any enhancing effect of omalizumab on IgE production. Results from the in vitro experiments indicate that omalizumab is not able to activate IgE‐producing cells by cross‐linking of the IgE‐BCR. This is in line with a study of Chan et al investigating the effect of omalizumab on the presence of IgE^+^ B cells and Cε germline transcription but not IgE production in vitro as we did.^34^ They observed even a slight decrease in IgE^+^ B‐cells numbers upon addition of omalizumab to the cultures. Limitations of our study include that only a limited number of patients and only 1 administration schedule were studied in vivo and regarding the in vitro experiments that the IL‐4/anti‐CD40‐switched IgE‐producing cells may not exactly mimic IgE‐BCR containing memory B cells. However, both the in vivo and the in vitro experiments did not provide evidence that omalizumab induces IgE production through activation of IgE‐producing cells.

The other hypothesis for the increase in IgE upon omalizumab administration which has been proposed recently suggests that complex formation between IgE and omalizumab, a human monoclonal IgG_1_ antibody, prolongs the serum half‐life of IgE by preventing its degradation utilizing the recycling of the complex by the neonatal receptor for IgG, FcRn.^15^, ^36^, ^37^ The latter hypothesis is difficult to test in allergic patients because it would require the administration of labelled IgE‐omalizumab immune complexes and a subsequent analysis of the half‐life of IgE as well as tracing of the complexes in vivo at an ultra‐structural level. We therefore performed an analysis of sera from subjects who had received s.c. omalizumab or placebo administration^24^ regarding allergen‐specific IgE levels to a large number of allergens using microarray technology^25^ (data not shown) and quantitative ImmunoCAP measurements. We found that the administration of omalizumab increased IgE to all allergens the patients was already sensitized to but no relevant new IgE sensitizations were noted. Only in 3 patients, IgE sensitization to cat and mite allergens exceeding cut‐off IgE levels was noted. This observation is in line with a recent study conducted by Mizuma et al., where the authors described patients while exhibiting Japanese cedar‐specific IgE levels above 0.35 kU/L only after omalizumab therapy had already suffered from cedar pollinosis before therapy.^13^, ^14^ Using an assay able to distinguish IgE‐omalizumab complexes from IgE and omalizumab alone, we could demonstrate the formation of IgE‐omalizumab complexes after subcutaneous omalizumab administration, which correlated to some extent with the increase in total IgE. These results indeed suggest that the rise in total IgE levels upon omalizumab administration is most likely due to IgE‐omalizumab complex formation resulting in prolongation of the otherwise relatively short half‐life of IgE.^15^, ^38^, ^39^

In summary, our results indicate that increases in IgE levels under omalizumab treatment are not due to cross‐linking of the BCR on IgE^+^ memory cells causing increased IgE production but rather due to formation of nonallergenic omalizumab‐IgE complexes with prolonged serum half‐life.

## CONFLICTS OF INTEREST

Rudolf Valenta has received research grants from Biomay AG, Vienna, Austria; Thermofisher, Uppsala, Sweden; Fresenius Medical Care, Bad Homburg, Germany; and Viravaxx, Vienna, Austria, and serves as a consultant for these companies. Dr. Marth reports personal fees from Novartis, personal fees from Thermofisher, personal fees from Teva, personal fees from Medmedia, non‐financial support from Boehringer Ingelheim, personal fees from Chiesi, outside the submitted work. Dr. Valenta reports grants from FWF, during the conduct of the study; grants and other from Biomay, grants and other from Viravaxx, outside the submitted work. Dr. Philippe reports grants and personal fees from Novartis, Roche, Genentech, during the conduct of the study. Dr. Lupinek reports personal fees from Thermo Fisher Scientific, outside the submitted work. Dr. Selb reports grants from Austrian Research Fund (FWF), during the conduct of the study. The other authors declare that they have no conflicts of interest.

## AUTHOR CONTRIBUTIONS

RV and VN designed and planned this study, participated in the interpretation of the findings, wrote, read and critically revised the manuscript. JED contributed to design and planning of the study, performed the data analyses, participated in the interpretation of the findings and wrote the initial manuscript and read and revised the final version. RF, RK, CL and TP performed measurements for specific and total IgE levels (CAP and CHIP measurements), read and revised the manuscript. PG and IM performed RBL assays, and KB and PV conducted basophil activation assays, read and revised the manuscript. RS, KM, RC, PZ, AM, KG assisted in planning and performing the intranasal challenge study, read and revised the manuscript. PhG kindly provided sera for analysis of subjects having received subcutaneous administration of omalizumab, read and revised the manuscript. IS and JG performed statistical analyses, read and revised the manuscript.
