# Supplementary figures and images for: Intranasal administration of allergen increases specific IgE whereas intranasal omalizumab does not increase serum IgE levels—A pilot study
Source: Allergy. 2017 Dec 12;73(5):1003–12. doi: 10.1111/all.13343 (PMC5969304; doi:10.1111/all.13343)

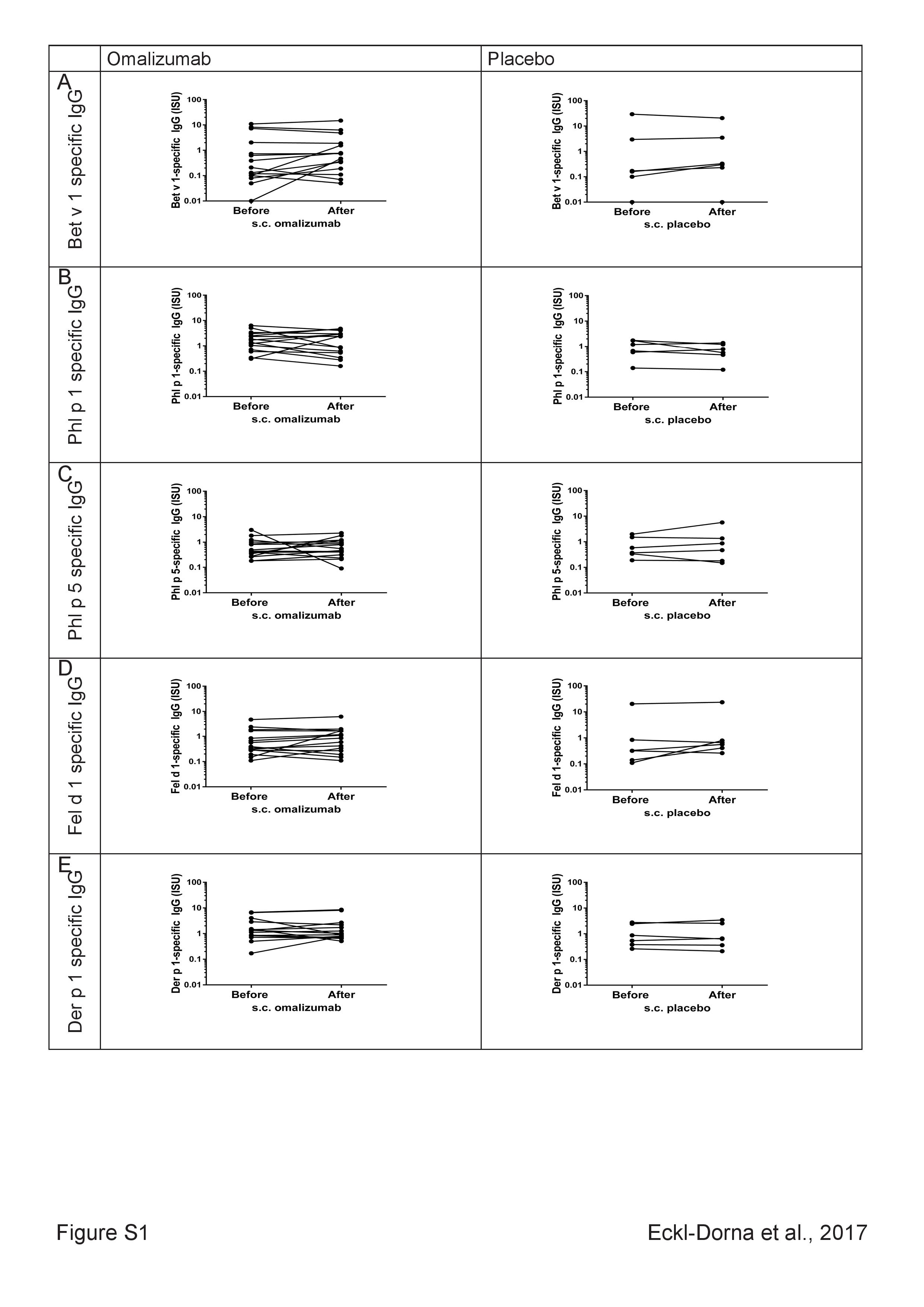

Supplement: Supplementary file 1 [file ALL-73-1003-s001.jpg]

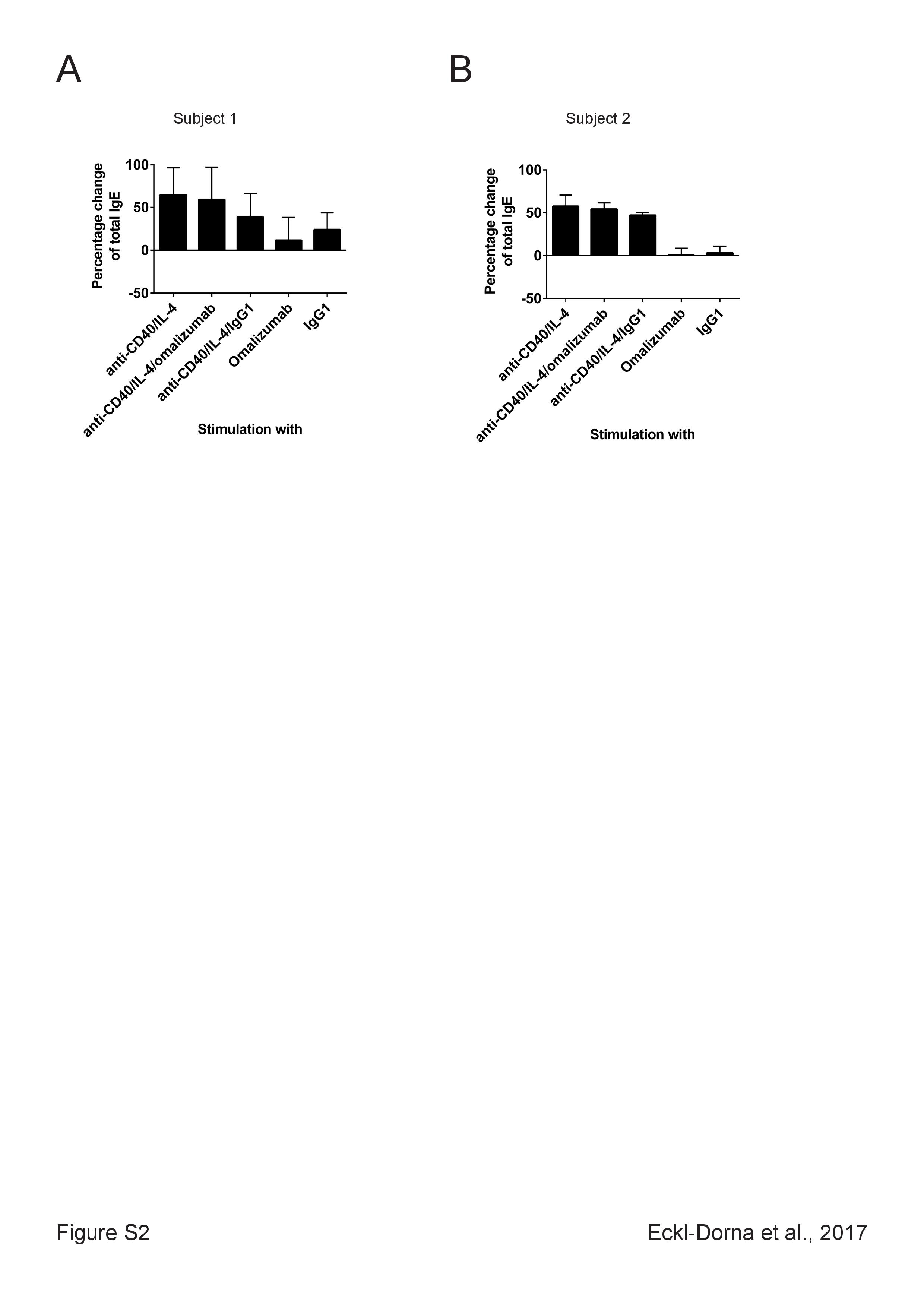

Supplement: Supplementary file 2 [file ALL-73-1003-s002.jpg]

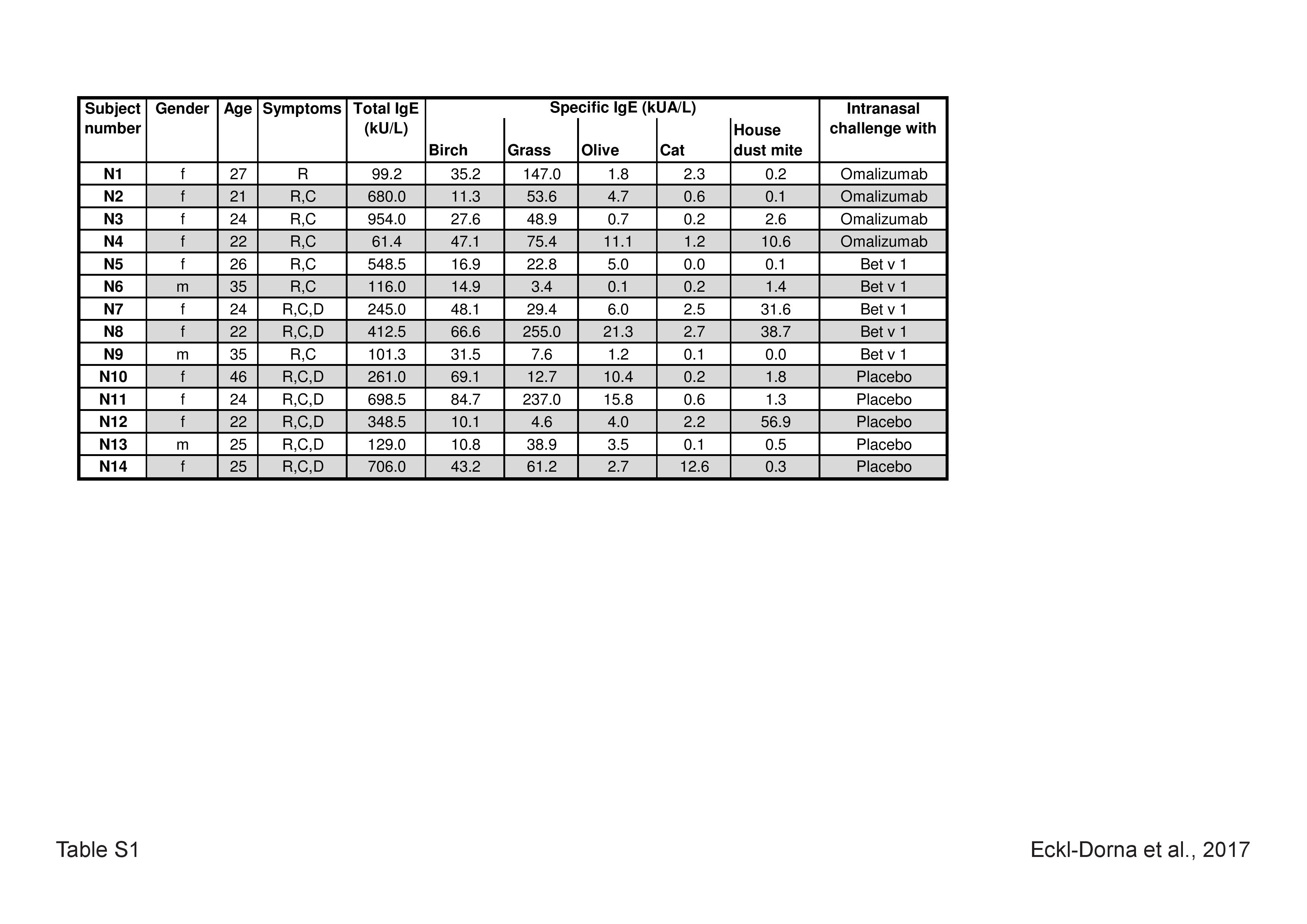

Supplement: Supplementary file 3 [file ALL-73-1003-s003.jpg]

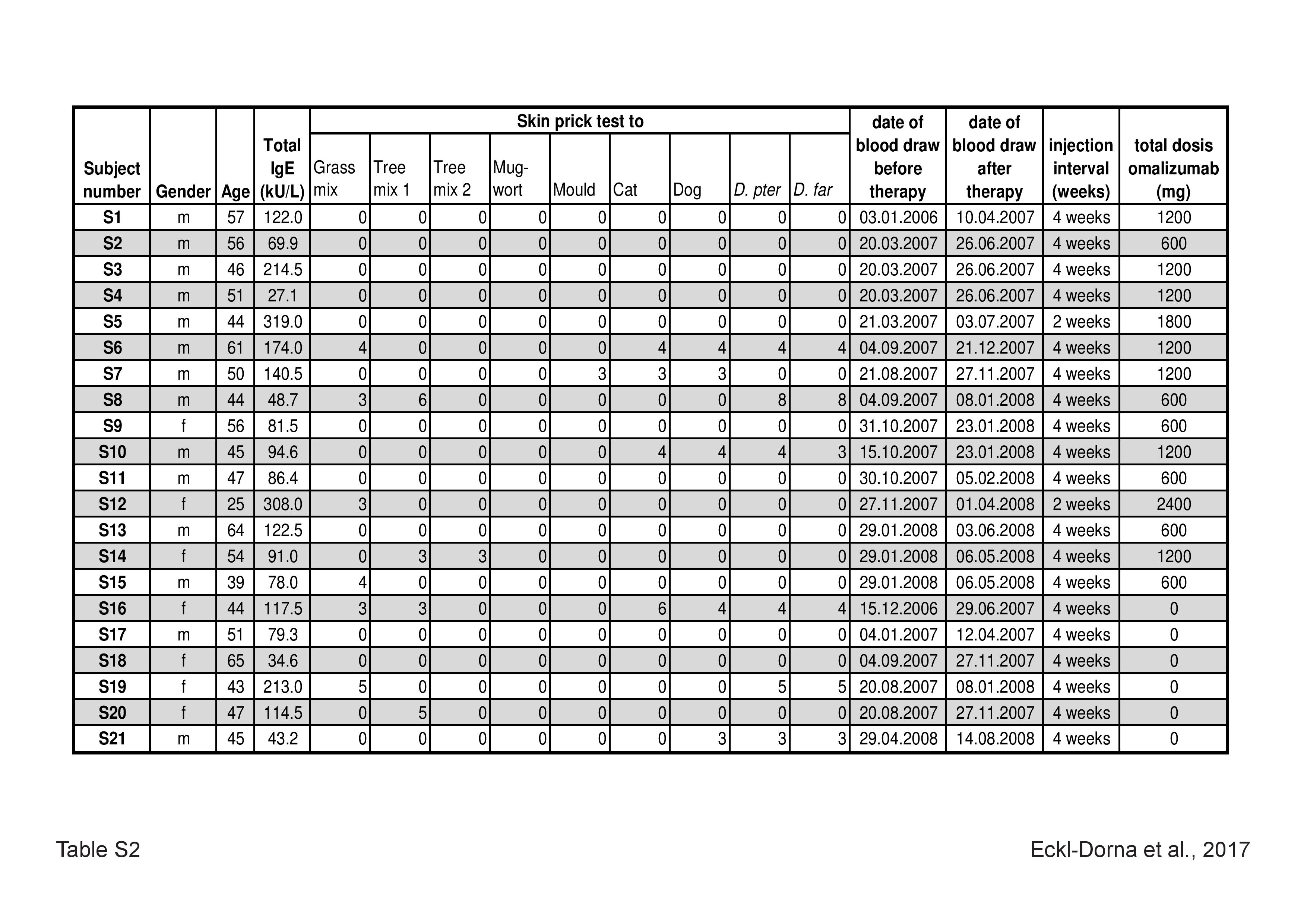

Supplement: Supplementary file 4 [file ALL-73-1003-s004.jpg]

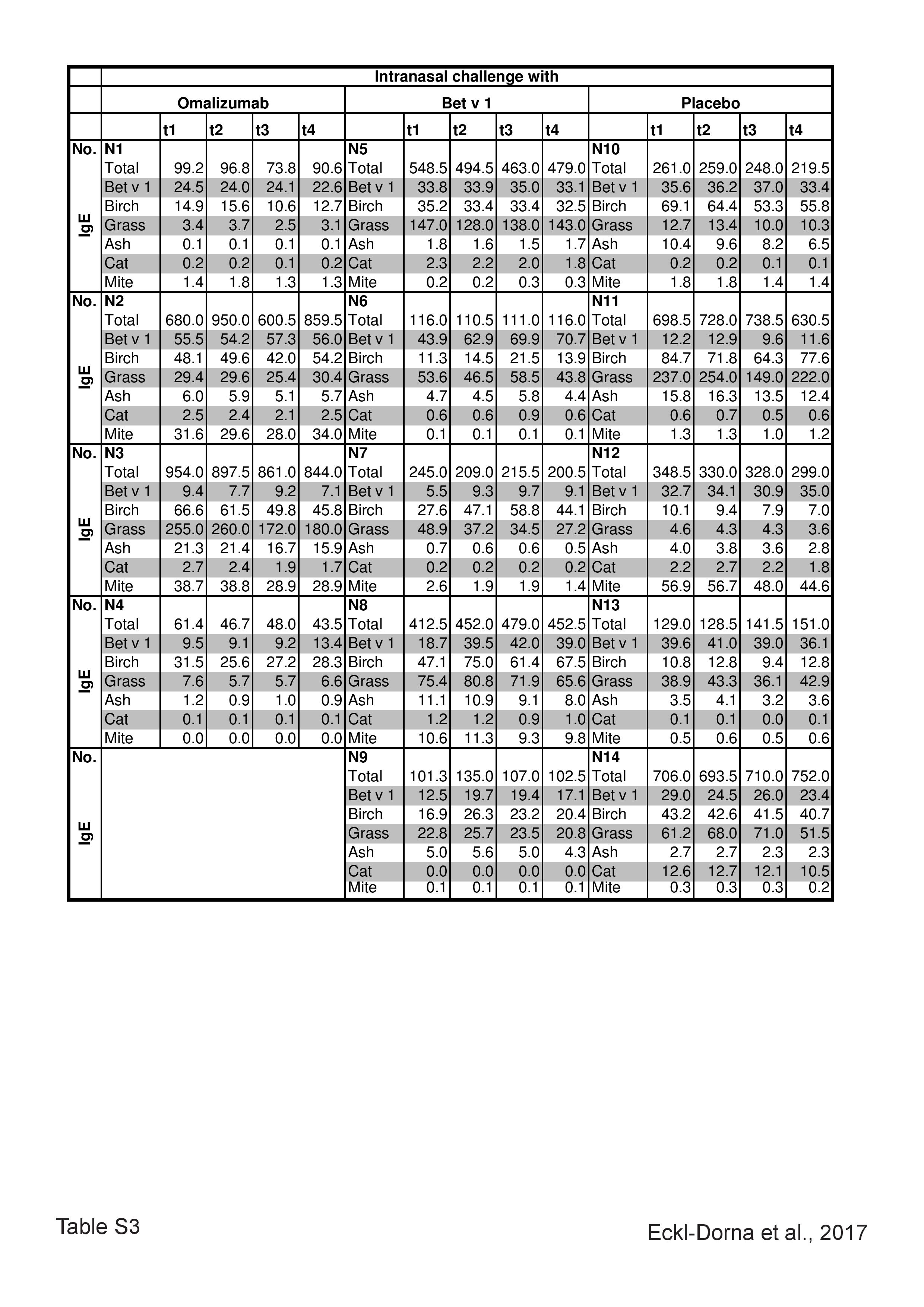

Supplement: Supplementary file 5 [file ALL-73-1003-s005.jpg]

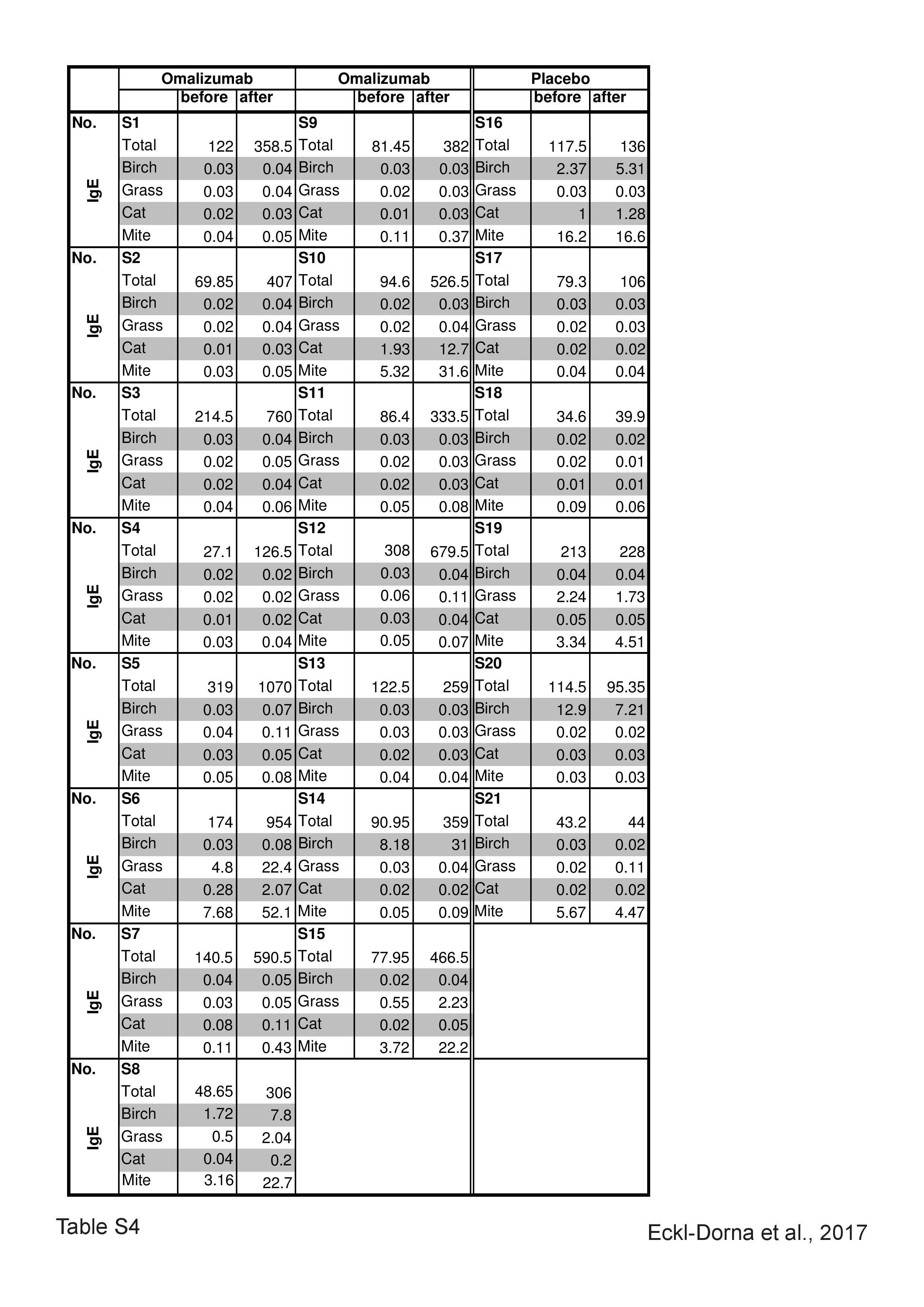

Supplement: Supplementary file 6 [file ALL-73-1003-s006.jpg]

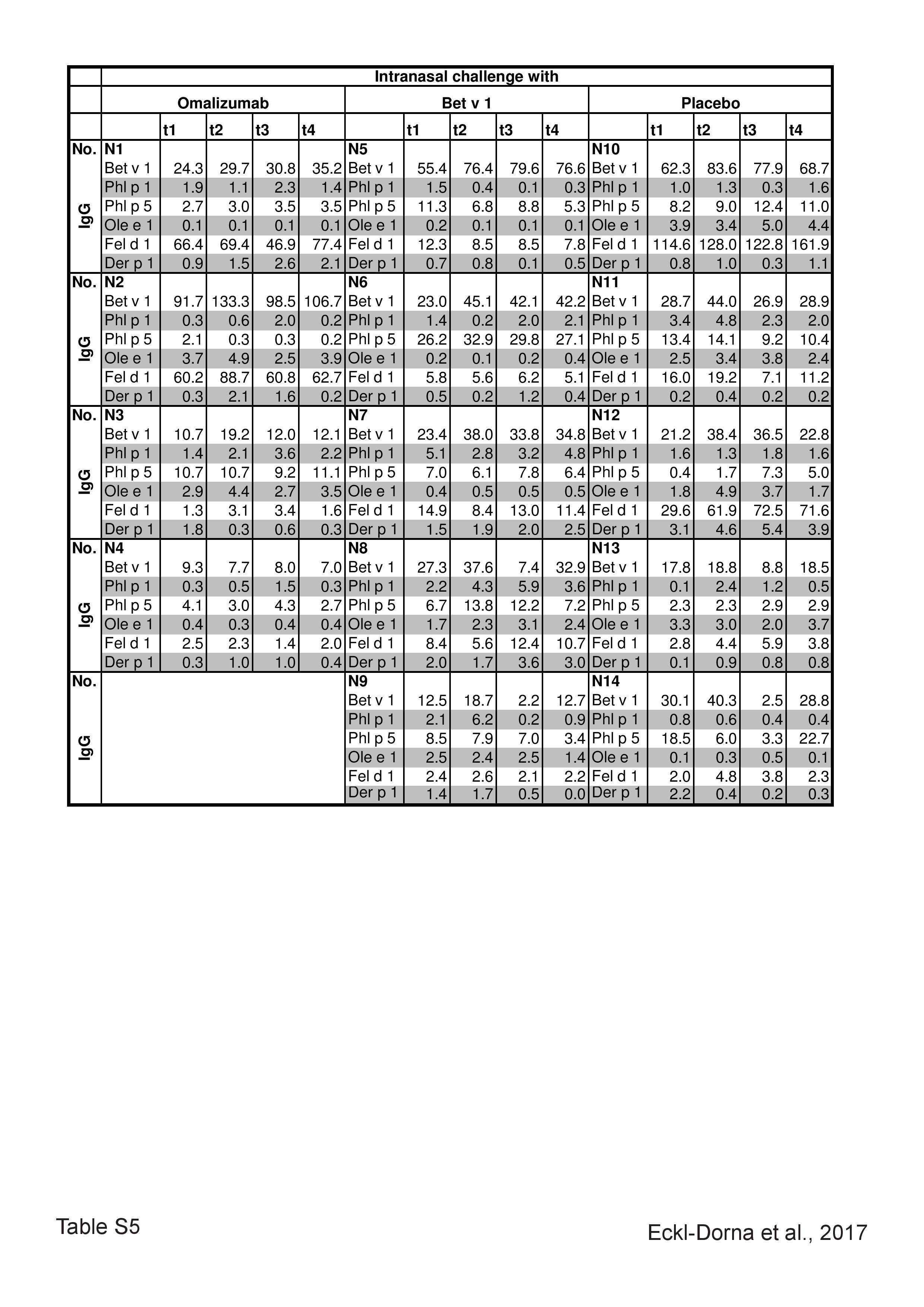

Supplement: Supplementary file 7 [file ALL-73-1003-s007.jpg]
