# Supplementary material for: Intranasal administration of allergen increases specific IgE whereas intranasal omalizumab does not increase serum IgE levels—A pilot study
Source: Allergy. 2017 Dec 12;73(5):1003–12. doi: 10.1111/all.13343 (PMC5969304; doi:10.1111/all.13343)
Supplement: Supplementary file 8 [file ALL-73-1003-s008.docx]

**Supporting Information**

**Figure legends**

**Table S1.** Demographic and clinical characterization of subjects who had been intranasally challenged with omalizumab, Bet v 1 or placebo. Gender: f: female, m: male; Symptoms: R: allergic rhinitis, C: allergic conjunctivitis, D: atopic dermatitis; kU/L: kilo units per liter, kUA/L: kilo units antigen per liter;

**Table S2.** Demographic and clinical characterization of subjects subcutaneously injected with omalizumab or placebo. Gender: f: female, m: male; kU/L: kilo units per liter;

**Table S3.** Total (kU/L) and allergen-specific (kUA/L) IgE levels of subjects intranasally challenged with omalizumab (left columns), Bet v 1 (middle columns) or placebo (right columns) as measured by ImmunoCAP in sera obtained at four different time points (t1: day 0, t2: day 21, t3: day 35, t4: day 56). N1-N14: subject numbering corresponds with Table S1.

**Table S4.** Total (kU/L) and allergen-specific (kUA/L) IgE levels of subjects before and after subcutaneous injection of omalizumab (left and middle columns) or placebo (right columns) as measured by ImmunoCAP. Before: before subcutaneous injection; after: after subcutaneous injection. S1-S21: subject numbering corresponds with Table S2. Sera “Before s.c. administration” were tested at visit V1 (V1: screening visit, at least 2 weeks before visit V2 where first dose of omalizumab was administered) except for subject S9 where serum was from V2. Sera “After s.c. administration” were tested at visit V8 (12 weeks after first subcutaneous administration) except for subject S18 where serum was tested at visit V6 (8 weeks after first subcutaneous administration) and for subject S16 where serum was tested at visit V10 (16 weeks after first subcutaneous administration).

**Table S5.** Allergen-specific IgG levels of subjects intranasally challenged with omalizumab (left columns), Bet v 1 (middle columns) or placebo (right columns) as measured by ISAC chip in sera obtained at four different time points (t1: day 0, t2: day 21, t3: day 35, t4: day 56) are displayed in international standard units (ISU). N1-N14: subject numbering corresponds with Table S1.

**Figure S1.** Allergen-specific IgG levels before and after subcutaneous administration of omalizumab or placebo. Levels of IgG (ISU, y-axes) specific to (A) Bet v 1, (B) Phl p 1, (C) Phl p 5, (D) Fel d 1 and (E) Der p 1 were measured by ISAC chip in sera obtained before and after subcutaneous administration of omalizumab (n=16) (left panels) or placebo (n=6) (right panels). Sera “Before s.c. administration” were tested at visit V1 (V1: screening visit, at least 2 weeks before visit V2 where first dose of omalizumab was administered) except for subject S9 where serum was from V2. Sera “After s.c. administration” were tested at visit V8 (12 weeks after first subcutaneous administration) except for subject S18 where serum was tested at visit V6 (8 weeks after first subcutaneous administration) and for subject S16 where serum was tested at visit V10 (16 weeks after first subcutaneous administration).

**Figure S2.** *In vitro* stimulation of peripheral mononuclear cells (PBMCs) with omalizumab in the presence or absence of anti-CD40 antibody and IL-4. PBMCs from 2 allergic subjects (A: left; B: right) were either left unstimulated or stimulated with omalizumab (3 µg/ml) or a human IgG_1_ antibody as control (3 µg/ml) in the presence or absence of anti-CD40 antibodies and IL-4. Supernatants were taken after 1 week of culture and percentage changes of total IgE levels compared to unstimulated cells (y-axes) as measured by ImmunoCAP are displayed. Means of triplicate experiments are shown. Error bars indicate standard deviation.
